# Understanding the potential role of Social Prescribing Link Workers in supporting identified needs of people with physical and mental long-term conditions: a qualitative study

**DOI:** 10.1186/s12875-025-02990-z

**Published:** 2025-11-19

**Authors:** Skaiste Linceviciute, Leire Ambrosio, David S. Baldwin, Mari Carmen Portillo

**Affiliations:** 1 Clinical and Experimental Sciences, Faculty of Medicine, NIHR Applied Research Collaboration Wessex, University of Southampton, 4-12 Terminus Terrace, Southampton, SO14 3DT UK; 2 School of Health Sciences, NIHR Applied Research Collaboration Wessex, University of Southampton, Building 67, University Road, Southampton, SO171BJ UK; 3 University Department of Psychiatry and Mental Health, University of Cape Town, Cape Town, South Africa

**Keywords:** social prescribing link worker, physical and mental long-term conditions, qualitative methods, community research

## Abstract

**Background:**

The Social Prescribing Link Worker (SPLW) approach is a means for supporting individuals and communities with diverse needs, with its reach and impact widely recognised in health and community systems. However, SPLW support for people with long-term physical and mental health conditions (P + MH LTCs) has been variable and there are knowledge gaps such as unheard voices of those with a varied engagement in SPLW support. We undertook a study to better understand the potential relevance of SPLW support for addressing the needs of individuals with P + MH LTCs. Its aim was to explore a range of health and psychosocial needs of people living with P + MH LTCs and to examine perspectives on how the SPLW role supports the complex needs of this group.

**Methods:**

A qualitative study utilising one-to-one semi-structured interviews with community dwelling adults (aged ≥ 18 years old) living with P + MH LTCs with diverse socio-demographic and clinical characteristics. Research was informed by a Patient and Public Involvement and Engagement (PPIE) group for meaningful and inclusive research activities, and qualitative data were analysed using a Framework Method.

**Results:**

Analysis revealed five themes and sixteen sub-themes that collectively demonstrate the complex and shifting experience of living with P + MH LTCs. This population dealt with competing multi-layered needs, and felt that the potential role of SPLW support to mitigate some of the unmet demands of this group was not effectively carried out in practice. This meant that potential benefits were often missed.

**Conclusions:**

Our findings demonstrate that this population is experiencing a substantial impact on health and wellbeing, and that there is an urgent need for integrated health and care systems that are complemented by consistent, coordinated and skilled SPLW support. Lessons learnt in this research provide new evidence and suggest directions for further research.

**Supplementary Information:**

The online version contains supplementary material available at 10.1186/s12875-025-02990-z.

## Introduction

Approximately 14 million people in England alone live with multiple long-term conditions (MLTCs) [1], and there is a predicted 34% increase by 2049 [2]. MLTCs can comprise physical and mental health conditions (P + MH LTCs) in the same individual [3], and have a bi-directional relationship, with a complex burden and adverse health and quality of life outcomes [4, 5]. The growing prevalence and increasing personal and collective burden of MLTCs is becoming a major public health concern, in the UK and worldwide [6, 7].

The coexistence of P + MH LTCs is associated with several sociodemographic factors including ageing, gender, and lower socioeconomic status (particularly for individuals living in the most deprived areas and communities, and/or facing unemployment), and also linked to lifestyle choices such as tobacco and alcohol use, poor physical activity and environmental exposures[8]. These complex social and cultural determinants and the demands of managing MLTCs together result in a greater use of healthcare services, many of which are not adequately equipped to support non-medical needs and challenges [9–11]. It is a continually shifting landscape featuring competing demands and priorities, and affected patients require sustainable, effective and relevant support [12–15].

A ‘social prescribing’ approach has been presented as a means for addressing a range of non-medical, socioeconomic and health related needs in people with LTCs, through a *‘community referral’* [16, 17]. Social prescribing is a mechanism that involves bridging health and social care services through partnership with voluntary and community structures to connect patients to local non-clinical services, so supporting them with a range of psychosocial and practical needs [18, 19]. Embedded in primary care or community settings, it is typically facilitated by a ‘Social Prescribing Link Worker’ (SPLW) who co-designs a personalised and meaningful social prescription based on a ‘what matters to you discussion’ and for the type of support thought to be needed [20–23].

Evidence around social prescribing for supporting individuals with LTCs has grown steadily [24–28], including its reach and positive impact in the wider community [29, 30]; particularly for studying patient and system-level outcomes including mental health, lifestyle, ‘belonging’ and healthcare utilisation [31]. However, systematic reviews have demonstrated that the way in which social prescribing is conceptualised, implemented, assessed and evidenced is highly variable, with limitations and gaps in knowledge [32–36].

A recently published book on *‘Social Prescribing Policy, Research and Practice’* made up of evidence and lessons learnt by leading researchers in the field called for comprehensive evidence expansion, some of which relates to the need for better understanding of cohorts that engage or do not engage with social prescribing. This includes but is not limited to, reasons and behaviours around those processes to ensure that social prescribing programmes, and particularly Link Workers delivering it, are appropriately equipped to support those in most need [37–39]. The authors pointed out that there are inconsistencies in access to social prescribing and a lack of clarity in how social prescribing support is configured to meet the needs of certain groups [38, 39], such as wide-ranging patients with LTCs [25, 26, 40–42]. This particularly concerns individuals with P + MH LTCs who share unique challenges and need adequate support but also have a varied engagement in SPLW support [36]. Studies predominantly report the experiences of already-engaged individuals [43] which can perpetuate the knowledge gap and leave the voices of non-engaged groups or those with variable awareness unheard. Given that one of the key priorities for social prescribing initiatives in the NHS 2019 Long Term Plan is to support patients with chronic illnesses and to address their complex needs [23, 44, 45], but also to encourage active engagement of local cohorts in social prescribing opportunities [46], it is therefore important to work with this population to understand how SPLW initiatives can support local individuals with P + MH LTCs in addressing their needs. Informed by this knowledge gap, we undertook a study to better understand the potential relevance of SPLW support for addressing the needs of individuals with P + MH LTCs. This involved exploring the lived experience of adults with P + M LTCs to understand their complex needs and examining how SPLWs’ role is equipped to address that experience. This was addressed through the following research questions:

1. What are the range of health and psychosocial needs of people living with P + MH LTCs?
2. What are people’s perspectives about Social Prescribing Link Workers’ role in supporting people with P + MH LTCs?

## Methods

### Design

This qualitative study was part of a larger project that aimed to determine the enablers and barriers to successful role of the SPLW support for addressing the complex needs of adults living with P + MH LTCs [47] and consisted of several work packages. In this paper we report findings from a qualitative study which utilised a framework method [48] that enabled a flexible yet rigorous approach to managing the data. In this study we were interested in exploring people’s experiences and perspectives. The approach has supported our focus on enabling an explorative development of subjective data with a meaningfully structured approach to analysis to bring knowledge on the subject under investigation. For comprehensive reporting of the qualitative study, the Consolidated Criteria for Reporting Qualitative Research (COREQ) guidance was used [49].

In this study we acknowledge the internationally accepted conceptual and operational definitions of social prescribing [50] while recognising that within the UK context, the use of social prescribing definitions can vary and adopt principles set out by the NHS England social prescribing model developed with varied stakeholders like the National Academy for Social Prescribing [51], and include the social prescribing link worker (SPLW) workforce development framework [23]. Thus, given the dominance of these principles within the UK context, the study has adopted the nationally used definitions/explanations of social prescribing.

### Patient and public involvement and engagement

Central to this study was a representation from the Patient and Public Involvement and Engagement (PPIE) group to ensure that all aspects of the investigation were empowered and guided by the voices of those affected with the issues examined in the study. PPIE members were involved in research activities around: a) interview schedule development, to ensure that questions were meaningful and relevant to individuals living with P + MH LTCs; b) provision of advice and reflections on data analysis to ensure that themes were representative, well-understood by lay public and demonstrated trajectory for impact; and c) provision of advice on recommendations, which could inform and guide SPLWs’ work in supporting patients’ needs with P + MH LTCs. To ensure a rigorous process of working with PPIE groups, the NIHR PPI resources were followed and guided our activities [52–54].

### Participant selection and recruitment

A purposeful sampling strategy [55, 56] was adopted to recruit individuals with diverse demographic characteristics that can offer diverse experiences and insights about the matters under investigation. This was complimented by a snowballing strategy [57, 58] that encouraged participants to discuss the study with their family, friends and other potentially relevant contacts. In the context of community based groups, the study advert was shared with existing member networks inviting participation and re-sharing with others who may find this relevant. As part of involving individuals from diverse backgrounds, we recruited individuals from areas and communities that may be more disadvantaged and under-represented, and often described as ‘difficult to engage in research’ considering ethnicity, socioeconomic status, vulnerable conditions, and geographical reach [59, 60].

Inclusion criteria were: a) being adults (aged ≥ 18 years old) with at least one physical long-term condition (e.g., including but not limited to diabetes, arthritis, asthma, COPD, hypertension) with a coexistent diagnosis of depression and/or anxiety; and b) being from local communities living in and around Hampshire, United Kingdom to inform research and guide support across the Wessex region. We excluded adults with mental disorders that were not depression and/or anxiety (e.g., schizophrenia, other psychosis), those who could not provide consent for themselves due to mental and/or cognitive capacity related problems, and those who were not able to communicate or understand the English language.

Advice on ‘data saturation’ in qualitative research is variable, thus we paid attention to previous empirical evidence in the field of social prescribing but also considered data adequacy for this study [61, 62]. A sample size of 20–25 participants was thought necessary given the varied understanding about social prescribing [63, 64], and for exploring diverse experiences from participants with marked variation in demographics and conditions, and for sufficiency of data to support themes that are being developed.

Recruitment took place through existing links and by building new connections with Voluntary, Community and Social Enterprise (VCSE) organisations, networks and groups, including but not limited to, Raising Voices in Research through Action Hampshire, So:Linked, Community First, Mind, Mental Health Foundation, local food bank, local Men’s Sheds groups, Southeast Thriving Communities (part of the National Academy for Social Prescribing), Restore Working for Mental Health, and other local community groups. This was achieved through appeals on social media and through established communication streams provided by NIHR ARC Wessex that promoted the study with their partners in regional third sector organisations, VCSE organisations, networks and groups.

The strategy of recruiting the population in question through active community engagement and involvement is integral for achieving relevant, robust and effective research that is meaningful to directly affected local communities [65, 66].

### Methods of data collection and setting

Semi-structured one-to-one interviews were conducted between May 2023 and October 2023. On average interviews lasted 50 min (from 22 to 138 min) and were conducted via telephone or via online video call (with or without a video streaming, depending on participants’ choice) in a confidential and quiet environment. Interviews were audio recorded, transcribed *verbatim* by a professional transcribing service, and anonymised to protect participants’ identities. As a remuneration for participants’ time and experiences shared, gift vouchers were offered.

Semi-structured interviews offered flexibility for modifying the interview schedule to suit the flow of interview, and the choice of using one-to-one interviews promoted a feeling of safety that supported richer and more open responses [55, 67].

Interviews were guided by an interview schedule informed by previous insights which emerged in our systematic literature review [36]. This was co-developed together with the research team and PPIE representatives, particularly those living with P + MH LTCs, who approved the interview schedule and other relevant accompanying documents. The research team had considered the complexity of this topic and the challenging nature of living with P + MH LTCs, thus the interview schedule used friendly and approachable lay language, to ensure that participants would not feel excluded by the use of academic terms, and that questions were meaningful and not intrusive.

The interview schedule included two main topic areas with additional probes on understanding the needs and experiences of living with P + MH LTCs and understanding the types of support for better living with P + MH LTCs (Appendix 1). Interviews were carried out by an experienced researcher (SL) with expertise in conducting in-depth qualitative interviews on sensitive topics with adult populations and the capacity to manage heightened emotional responses. Additionally, post-interviewing notes were taken and reflections were discussed with DSB through supervision. No participant withdrew from the study. Non-participation rates are presented in Table 1.

**Table 1:** Participants’ socio-demographical and clinical characteristics

Description			Total (%)
Age	20–29		3 (13%)
	30–39		3 (13%)
	40–49		5 (21%)
	50–59		9 (39%)
	60–69		1 (4%)
	70–79		2 (8%)
Ethnicity	White		16 (69%)
	Ethnic Minority		7 (30%)
Gender	Women		14 (60%)
	Men		9 (39%)
Employment	Unemployed		2 (8%)
	Part-Time		9 (39%)
	Full-Time		9 (39%)
	Retired		3 (13%)
Marital Status	Married		9 (39%)
	Single		11 (47%)
	Cohabiting		3 (13%)
Education Level	Secondary Education		11 (47%)
	Higher Education (Undergraduate)		5 (21%)
	Higher Education (Postgraduate)		7 (30%)
Living Circumstances	Living Alone		7 (30%)
	Living with Someone Else		16 (69%)
Physical LTCs	Bowel	Irritable Bowel Syndrome	1 (4%)
	Chronic Pain	Fibromyalgia and Other	8 (34%)
	Gynaecological	Endometriosis and Other	2 (8%)
	Heart	Chronic Cardiovascular Diseases	4 (17%)
	Metabolic	Diabetes and Other	7 (30%)
	Neurological	Chronic Fatigue Syndrome	4 (17%)
		Epilepsy	3 (13%)
		Migraine	1 (4%)
		Movement related Disorders	6 (26%)
	Respiratory	Asthma	2 (8%)
	Rheumatoid	Arthritis and Other	4 (17%)
	Sensory	Sensory impairment	2 (8%)
	Thyroid	Other	1 (4%)
	Other	Long-Covid	1 (4%)
Mental LTCs		Depression	18 (78%)
		Anxiety	11 (47%)
Numerical overview of P + MH LTCs	Participants with at least 1 P LTC together with depression and/or anxiety		11 (47%)
	Participants with 2 P LTCs together with depression and/or anxiety		8 (34%)
	Participants with 3 P LTCs together with depression and/or anxiety		1 (4%)
	Participants with 4 P LTCs together with depression and/or anxiety		2 (8%)
	Participants with 5 P LTCs together with depression and/or anxiety		1 (4%)
Non-participation	Not met inclusion criteria		6 (26%)
	No response after receiving participation documents		2 (8%)
	Not answered on the day of the interview and not followed up		2 (8%)
Participants awareness of SPLW support	Heard the term, but never engaged/been offered support or aware what it entails		9 (39%)
	Heard the term, engaged with support and found it useful		2 (8%)
	Not heard the term, but would be interested if ever offered		5 (21%)
	Tried SPLW support but it was unsuccessful		5 (21%)
	Not heard the term, unsure if it would be useful		2 (8%)

For contextual purposes, in Hampshire, England, SPLWs are embedded within Primary Care Networks (PCNs) as part of NHS England's integrated care approach [51]. This involves working within existing social prescribing infrastructure in both primary care and community-based settings, partnering with local agencies, third sector organisations and the NHS.

### Data analysis

Qualitative data (i.e., transcripts) were analysed using a Framework Method that sits within the broad family of thematic analysis and is widely used in multi-disciplinary health research [48]. This method consists of seven interconnected stages, designed for establishing a systematic identification of qualitative themes. Our analytical process comprised inductive and deductive components to ensure that we focused on the topic of investigation, guided by the key literature, project objectives and the input from PPIE, but also had space for novel meaning to emerge. Following the transcription and interview familiarisation steps, our analysis began inductively through unrestricted coding to form an insight into the range of aspects and impressions that were emerging through the data. A selection of transcripts was independently coded by two members from the team (SL and LA) to achieve a detailed understanding of the emerging labels that were then discussed and agreed on for further application to the remaining transcripts carried out by SL. Development of the analytic framework was an iterative process supported by members of the PPIE group to ensure their viewpoints were considered for a meaningful representation of the issues under investigation. Throughout this process, NVivo 14 qualitative data analysis software was used that supported systematic chartering of the data and was essential when navigating the interrogation and refinement of codes into broader themes. A list of five identified themes was reviewed by all members of the team and is presented along with representative quotes.

### Reflexivity

The research team was made up of four academic professionals with different backgrounds and expertise. All authors had expertise and necessary training for undertaking qualitative research and for working with adult populations that may be affected unequally by their health needs. As academic research team we had a shared interest in community based support systems like social prescribing, and wanted to establish a better understanding of the social prescribing model involving Link Workers and how it may be equipped to support the needs of people with P + MH LTCs. This collaborative approach was judged necessary to achieve study objectives and to expand knowledge that could inform our team’s academic and clinical expertise for further work in the field. As a research team we were diverse in our socio-demographic characteristics such as age, gender and cultural backgrounds, which have enriched our conversations and offered differing perspectives.

As for participants, the team members were not known to participants prior to data collection. None of the participants raised concerns about the research team or the study itself; instead participants were pleased to know the research was being done.

## Findings

### Participants characteristics

Twenty-three participants were recruited in the study and their socio-demographical and clinical characteristics are shown in Table 1. Our study sample consist of diverse socio-demographic characteristics, and participants also had varied physical conditions alongside depression and/or anxiety. Importantly, there were a few participants who described some of their conditions as rare: therefore, where applicable those conditions were included within broader applicable disorder categories to protect participants’ anonymity and to ensure that the reader is still presented with reliable information. As participants held different awareness about SPLW support, these insights are presented in numerical description to offer greater credibility to the findings, and are shown in Table 1. It is important to note that these responses are part of subjective interview data and have not been gathered using standardised measures.

### Themes and sub-themes

**Fig. 1:**
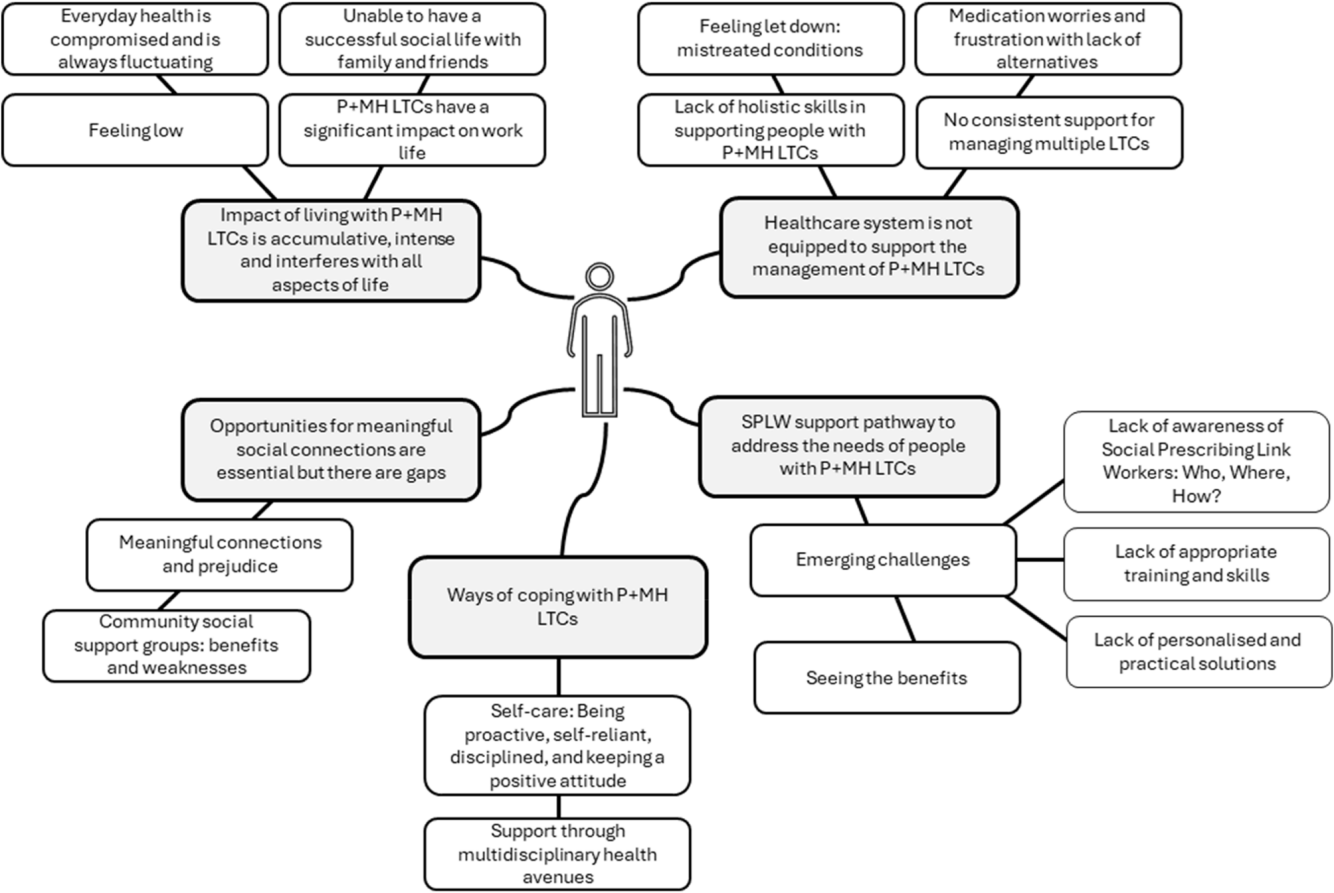
Qualitative themes and sub-themes representing the data

Five themes and sixteen subthemes were developed (Fig. 1) from analysing the interviews with adults with P + MH LTCs. These illustrate a range of experiences around health and psychosocial needs of living with P + MH LTCs and highlight diverse perspectives on the potential relevance of SPLWs’ role in supporting participants’ needs. Themes are provided with illustrative quotes, and more detailed information about participants is presented in Additional File 1.

#### Theme 1. Impact of living with P + MH LTCs is accumulative, intense and interferes with all aspects of life

This theme is made up of four sub-themes that discuss the cumulative impact of P + MH LTCs on people’s mental wellbeing, functional status, participation in social life and employment related matters. Effectively, participants expressed context-specific concerns.

1. Everyday health is compromised and is always fluctuating

For most participants living with P + MH LTCs was life-limiting and burdensome*.* Collectively, participants emphasised a prominent sensation of pain and physical discomfort accompanied by exhaustion, ‘brain-fog’ and overall deterioration in physical and mental performance that compromised their life quality:

*“…so fatigue is the major impact on my life and pain. I get a lot of pain with it and a brain-fog. I can't think as well, and I have to try and compensate for that. So you feel that you haven't gone… In your life, you've not done as much as you would like to have* < *..* > *I find it absolutely exhausting” (P1)*

While some participants managed to experience ‘cheerful’ and energetic days and were able to achieve *“goals for that day” (P20),* most participants were never completely free of their symptoms, instead symptoms varied in severity and frequency. Fluctuating and often unpredictable symptoms had a ‘cumulative effect’ and became interlinked*.* In turn, on ‘bad symptoms days’ participants were unable to undertake domestic, or in some cases basic self-care related tasks*,* general mobility would deteriorate, worrying would exacerbate with feelings of withdrawal*.* Evidently, this was a result of having to ‘constantly adjust’ and accept that their bodies are unable to cope with what once was ‘normal’. This loss of control was defeating for many:

*“…life gets quite tedious sometimes* < *..* > *there's so many things that I'd like to do in my mind, that my body can't do, that my body won't allow me to do* < *..* > *I focus on something, and, again, it's part of trying to make myself better. Yes? Then I'm let down by my body.” (P9)*

2. Feeling low

Beyond the day-to-day impact of having MLTCs, over one-third of participants suffered with poor mental health. Participants shared moments when depressive symptoms were prominent, often resulting in total disengagement and heightened emotions of sadness, feeling ‘miserable’ and ‘sunken’, inadequate or ‘totally humiliated’. For others this was experienced through more complex emotions like anger and behaviours such as seeking risky social interactions or contemplating suicide and questioning their life quality and worth in society:

*“I am left in a lot of pain, and I am more disabled now than I've ever been* < *..* > *Some days I don't want to get out of bed. Some days, yes, the sun is shining so I'm better, but some days I don't want to be here. I don't want to be a burden on my family.” (P10)*

Although the severity of such distressing feelings varied, few participants required support from mental health specialists and in-patient care to help manage overwhelming symptoms. Those encounters were stressful and worrying, and devastating for families watching it unfold:

*“That depression, I don't want to go back to the hospital because of it* < *..* > *I'm thinking a lot about the family. The children are left behind. My husband. I think a lot of the house,* < *..* > *my future. Am I going to be all my life like that, to be sectioned*^*1*^* all the time? It makes me worry enough. Am I going to die early and leave my children and my husband?” (P22)*^1^

Evidently participants were grieving a range of losses because of having to live with life-impairing LTCs. But more importantly, participants were faced with uncertainty and fear for future exacerbations which suggested minimal to no planning in place for supporting mental health.

3. Unable to have a successful social life with family and friends

Socialising was another aspect affected by P + MH LTCs. Having MLTCs meant that some participants had to take precautions such as eliminating triggers that can exacerbate their conditions, thus having reduced social contact with family and friends. For example, participants felt the need to decline invitations to social gatherings, eliminate participation in physically-exerting activities and in some cases prioritise time alone, particularly when feeling unwell and ‘overstimulated’:

*“In terms of social situations, if I'm ill, if I'm physically ill, I tend to avoid them. I tend to just kind of stay on my own a lot, which can feel quite isolating. I know that it's for the good of my health, to stop me picking up any infections, and to keep me kind of doing okay.” (P3)*

In turn, this has come at a cost of lost connectivity to others and increased social isolation. Some participants believed it was their lack of social skills in building friendships and social networks or having mental health issues and being ill as barriers to social connections. Others felt it was the lack of empathy in people for not recognising participants’ ‘suffering’:

*“just the energy where I let people down, where I've said yes to something. So a friend's now decided not to speak to me because I wasn't able to go to something a few weeks ago, and that makes me so angry* < *..* > *because of their lack of understanding, 40-year friendships gone” (P19)*

Social isolation was ‘emotionally upsetting’, when combined with symptoms such as exacerbated pain and depression, it became a reason for escaping in alcohol use. Although only a few participants have voluntarily brought this subject to attention, it highlighted the link between social isolation and other underlying issues such as alcohol use in people with P + MH LTCs:

*“Another big problem* < *..* > *is alcohol.* < *..* > *you're so depressed that you want a drink because the drink takes it away. In my case, it dulls the pain and it makes me forget,* < *..* > *lightens your mood* < *..* > *I got to the stage where I needed two bottles of wine, not one, but it meant that I could move around easily, better, I was in less pain and I slept better* < *..* > *it is something very embarrassing to admit to because you don't need it on prescription. Behind closed doors, nobody knows what you're up to” (P10)*

Altogether, participants’ reflections demonstrated the importance of needing compassionate social networks and companionship for supporting people in unpredictable living with P + MH LTCs.

4. P + MH LTCs have a significant impact on work life

Of 23 participants, 18 were in employment, and work-related impact was a significant concern to this group. A large portion were worried about disclosing their P + MH LTCs to work colleagues and managers. People were fearful of judgement and bias towards their capabilities, but also for being overlooked for promotions. For others, however, due to necessary adjustments such as having to arrive to work later, reducing working hours, and utilising options such as working from home, meant that participants felt the need to disclose and share their health struggles with their managers/supervisors. Although, there were a few participants who felt supported and as a result were given ‘tools’ such as flexible working pattern to help with the management of MLTCs, particularly when “*having a bad pain day” (P13)*, others did not receive a favourable outcome. Instead, they felt stigmatised, particularly when challenged for medical proof:

*“I was told to bring a medical proof, such as my diagnosis, it was something that was really getting me sick* < *..* > *I really felt they gave me conditions of being laid off or having to seek part-time basis and that I need to bring that medical report for my conditions. So that was when I felt it was necessary to open up to my company about my condition.* < *..* > *that really made me so stigmatised” (P21)*

Those participants highlighted the need for accessing anonymous occupational health support and for options to report bullying that they did not feel comfortable sharing with their superiors. Being made to feel that “*this is all in your mind, even though you know it isn't*” (*P12)* was distressing, particularly during the phase when diagnosis was not confirmed.

In addition to work-related stigma, participants shared concerns around reduced performance that affected quality of their work. Some participants felt it was necessary to take prolonged sickness leave or to end their career altogether due to combined impact from P + MH LTCs affecting concentration and causing errors:

*“So could barely walk. Aches, pains, brain fog. I wasn't fit for purpose anymore, for my job. So I wasn't able to actually go and do that work. I started making mistakes with medication. Basically, just my brain wasn't as sharp as it used to be.” (P19)*

Losing the ability to work and to utilise their professional skills exacerbated depression and affected people’s self-esteem. People agreed that most workplaces were not equipped (i.e., through resources and awareness) to support workers with MLTCs, and obstacles like stigma and lack of empathy created adverse effects.

#### Theme 2. Ways of coping with P + MH LTCs

Participants discussed the ways of coping with their conditions, and the analysis revealed two sub-themes that reflect their coping strategies, including self-initiated behaviours and attitudes with support from multidisciplinary health professionals and related avenues.

1. Self-care: Being proactive, self-reliant, disciplined, and keeping a positive attitude

Findings revealed that most participants reached a point in their journey with P + MH LTCs where they felt it was necessary to practice independence and take ownership in looking after themselves. Participants recognised that *“the [healthcare] system is so oversubscribed*” (*P4).* They admitted that individual responsibility and acting in managing their own health was important for building resilience and having sustainable support long-term.

In turn, participants self-initiated various coping methods for managing P + MH LTCs such as through physical activities and gentle movement like walking, diet monitoring, having a strict sleep schedule, taking pain relief when necessary, releasing frustration through swearing, playing a guitar, taking non-medical supplements and utilising non-medical therapy like acupuncture or meditation through digital applications, and trying out specialised equipment. For some participants, coping was also about keeping a positive attitude and focusing on their capabilities and achievements instead of losses:

“My energy a lot of the time is strong. It isn't always, obviously, but I have a tremendous desire to do something of value in the world still, and it doesn't stop me.” (P16)

Others were focused on health outcomes and thus were determined in achieving better health with P + MH LTCs. A lot of the time that meant pushing themselves to the limit and out of their comfort zone to achieve a desired outcome:

*“It is actually quite hard just sitting still talking to you,* < *..* > *I know if I don't do it, the mental dialogue of, 'I couldn't go there because of my dizziness,' or other, is not helpful. It's better to build up a history of managing to do things, even if they're hard.” (P12)*

Some however reflected on the importance of slowing down, resting and recovering, and knowing when to seek support. Participants agreed that managing your own health was a learning process, and that it was important to tune-in to their needs to understand what works and what needs improving. Effectively, this level of self-awareness required discipline like being mindful of triggers, monitoring change in health outcomes and having a structure to a daily life:

*“my health is pretty good, but I think because over the years I've learned to adapt my life or lifestyle, partly to not to exacerbate either my physical health problems or my mental health problem. So it's a daily structure and routine” (P4)*

Not all participants developed all the above behaviours and attitudes for coping with their P + MH LTCs. Some individuals were more self-aware and reflective than others, thus demonstrating diversity in attitudes and coping experiences. There was, however, a sense of consensus around the importance and urgency of learning to adapt to their LTCs, often through trial and error.

2. Support through multidisciplinary health avenues

In addition to proactive role of self-initiated coping strategies, a few participants were successful in securing access to diverse support avenues such as specialist centres or rehabilitation programmes guided by multidisciplinary teams and specialist mental health treatment led by highly skilled health workforce:

*“I've recently been under a new team at the hospital, so a specialist Endometriosis Centre* < *..* > *That's the first time I've ever really felt like I am getting the support I need from health professionals.* < *..* > *They approach it from more of a holistic point of view, so they want to deal with all of your symptoms, but also make sure that your mental health is okay” (P13)*

Although there has been no reference on the length of time and effort it has taken for participants to access and sustain these multidisciplinary support avenues, reflections suggest positive and beneficial outcomes for those who did. Appreciation was particularly expressed to mental health specialists who were encouraging in helping participants to navigate complex lives with MLTCs. Their reflections confirmed the importance of having supportive and multi-skilled healthcare team. However, only a handful of participants were able to experience an all-round support for their P + MH LTCs:

“although I didn't specifically see someone about chronic back pain at the time, where the anxiety was concerned I did <.. > Again, they look at your overall picture of what's going on” (P8)

#### Theme Three. Opportunities for meaningful social connections are essential but there are gaps

This theme is composed of two sub-themes linked to the importance of social connections and interactions as a source of support for helping participants manage diverse demands with P + MH LTCs. This theme entails both, positive and negative reflections about the importance of connecting and belonging to others.

1. Meaningful connections and prejudice

Participants with compassionate relationships with family and friends valued their support and shared examples of appreciating help with domestic tasks and general care needs, and were also sentimental about the motivational conversations and devoted attention that helped participants when feeling low:

“My family members help with my depression, no stress they say. They always love staying around me to keep me company, so I don't feel the depression much” (P23)

However, participants highlighted that meaningful social connections with family and friends needed to be built on trust and thoughtfulness to allow participants feel safe and supported. Not all participants experienced positive connections, there was some who encountered prejudice and stigma related to their MLTCs. Reflections revealed that participants were judged on their appearance, age, ethnicity and other factors in relation to what certain chronic conditions and symptoms should look like and who should have them:

*“…people don't understand, because they think you still look okay. So because I wear makeup and I like to dress nice, so they're like, 'Oh, if you're feeling that unwell, you wouldn't even be putting your makeup on.' It's just prejudice that I've come across.” (P19)*

Some participants had concerns that there was a general lack of awareness and interest from their family members, friends, acquaintances, but also healthcare professionals in understanding the impact or barriers of someone affected by P + MH LTCs. Participants reflected that insensitive comments, lack of empathy, and misinformed assumptions made them feel overlooked and isolated but also anxious of not belonging:

*“You're also socially isolated. You do try to join in sometimes* < *..* > *People say oh, you're a miserable devil, what are you doing sitting down there?* < *..* > *they're all standing with a plate and a glass, I can't even do that* < *..* > *you do get a lot of abuse. People don't understand. They look at you and say, 'Well, there's nothing wrong with you', but they don't see me standing. They see me leaning on a door or on a post* < *..* > *and then they say, 'Oh, you've had too much to drink'*.” *(P10)*

In turn, some become watchful over disclosing their health issues to fit in and feel ‘normal’. For others, this resulted in relationship and friendship breakdown. Similarly, a few participants from an ethnic minority group spoke about ‘bottling up’ as discussing wellbeing and mental health struggles was *“deemed as a position of weakness* <.. > *It's still a touchy subject until today. It's quite difficult for me to even speak to my mum about things like this”* (*P18*).

Although interactions with close social groups provided emotional, social and domestic respite, for others this was associated with insensitive comments and prejudice and a source of anxiety. Participants highlighted that they hoped for compassion and better understanding of what it means for someone to live and cope with P + MH LTCs.

2. Community social support groups: benefits and weaknesses

Findings demonstrated that beyond immediate family and friends’ support, participants were attracted to the idea of building meaningful connections with other people with LTCs through community based social support groups and similar avenues.

Evidently, there was a mixture of participants: those who have previously attended, were current members or were interested in pursuing social support through community-based groups. The purpose and the setup of those social support groups appeared diverse and ranged from café type social groups, church groups, small self-initiated gym network, local support groups stemming of national charities, to primary care and mental health services led support groups for patients. Effectively, drawing clear description regarding the nature and access to social support groups was difficult because participants were mostly concerned with the relevance and impact of groups.

Amongst enthusiasts who were encouraged by this avenue of support, reflections revealed several helpful and motivating reasons for attending. Social support groups were a platform to exchange ideas about coping strategies and learn how others work through their ‘daily challenges’, including an opportunity to pursue new skills from experts in the field. Furthermore, social support groups were a way of meeting and connecting with like-minded individuals, either with same or different LTCs and in turn, experience a sense of belonging:

*“it was a Godsend, to say the least, when she introduced me to this group* < *..* > *it's an avenue for me to interact with females like myself from all walks of life and just let our hair down and have fun as much as possible” (P18)*

Others added that social support groups offered a safe place to open up, and, for many others, it was the main outlet of support for their physical and emotional needs:

“I don't know where I'd be if it wasn't for that group, because I've got no support from my doctor, I've got no support from anybody. So that group is wonderful.” (P17)

Notwithstanding, not everyone felt comfortable with the setup of social support groups. Some participants indicated that social support groups promoted discussion around negative consequences of living with P + MH LTCs. The accentuated focus on challenges instead of successes was reinforcing ill-health identities and reducing belonging:

*“Actually, the problem with support groups* < *..* > *people, then, they define themselves as that problem or that issue* < *..* > *it can almost get to the point where it's reinforcing that you've got this problem and you, then, come to identify yourself as that. It then almost becomes even more difficult to integrate into a society where those problems aren't talked about.”* (*P14)*

Others however, pointed out that some social support groups, although well-intended, have promoted medical model instead of utilising biopsychosocial ideas and exploring reasons why people struggle to manage their chronic conditions:

*“I got introduced to a group* < *..* > *that was led by an inspiring guy who had diabetes, and, basically, cured himself through a mixture of diet and exercise.* < *..* > *I started to get irritated by the fact that he was doing the same thing as clinicians, like, 'You really do need to be eating the right things and exercising', and all that. That group actually spent very little time on things like vulnerability and failure.* < *..* > *it was almost like, only ever say anything positive, be positive all the time, only say what's working,* < *..* > *the irony is, the more I can talk about my failure in a group like that, the more successful I would probably be.” (P16)*

Beyond that, some participants were also concerned with practical arrangements that widened a disengagement gap, namely:

- online attendance reduced interaction: “*the group moved to Zoom, and I found the Zoom sessions weren't as good” (P5),*
- online resources excluded people without access or skills: *“I was never aware of any support groups* < *..* > *I don't really use social media, so I don't really see that.” (P13)*,
- financial costs affected attendance: *“ashamed to be not known of where you can get funding or support for that.” (P9),*
- ceased activities in smaller geographical locations: *“They've decided, well, we'll just abandon all the people in [name of location] because they* < *..* > *have an office* < *..* > *which is 40 min from here.* < *..* > *Unfortunately for us guys here, apart from one of the churches who does a get-together, there's not much that goes on.” (P5),*
- lack of age, gender and ethnicity specific social support groups that would enable sharing sensitive challenges with peers from similar backgrounds and those with similar experiences: *“I would like to stay with people that are also passing through the same thing, to share some meals together, and help each other” (P23).*

#### Theme 4. Healthcare system is not equipped to support the management of P + MH LTCs

This theme has four sub-themes linked to complex set of concerns around the UK’s NHS healthcare system that participants felt is not equipped for patients with multiple LTCs like P + MH.

1. Lack of holistic skills in supporting people with P + MH LTCs

Findings from the interviews suggest that holistic health care was not achievable for all. Participants disclosed that their P + MH LTCs were treated in isolation and on a priority basis, depending on which condition or symptom was critical and needed attention at the time:

“The biggest difficulty is if you have comorbidity <.. > that it's all—you're treated everything in isolation <.. > You're not treated as a whole person” (P2)

Participants were aware of the interplay between their MLTCs and felt frustrated that ‘root causes’ of one condition linked to another were ignored or downplayed: some provided support did not align to their sensitive needs like those related to male erectile dysfunction:

*“I was told, 'Well, you can't have a referral to a dietician because your scores aren't high enough. You can only have it if you're a fully blown diabetic* < *..* > *so I couldn't be referred, and it's the same with erectile dysfunction. I've given up on that. I don't even mention it to the doctors anymore, because what's the point, but it's related.” (P5)*

In fact, participants were disheartened with the lack of interest and empathy towards their struggles, particularly those related to mental health and other sensitive circumstances:

*“the nurse that I spoke to, and GP that I've spoken to about the condition, when I really opened up and started to get personal about the impact it had on me, I found their embarrassment. Now, that shut me down. As soon as I experience someone else's awkwardness or embarrassment, or limitations, it's like a freezing process, and I think I can't go anywhere here, it's not safe, so I shut down” (P16)*

Participants recognised that health professionals in primary care had time and target restrictions but were concerned with their poor understanding around the importance of supporting holistic needs of P + MH LTCs and addressing collective impact on patients’ wellbeing. People felt that support was ‘tokenistic’, and that advice was either ignorant such as to ‘take a drink’ or insufficient in contrast to the problem. This, combined with lack of skills in person-centred care, left participants with multiple unmet health needs.

2. Feeling let down: mistreated conditions

Findings revealed that there was a group of participants who in addition experienced stressful circumstances of mistreated P + MH LTCs. Evidently, these participants endured worrying situations when their health concerns were not taken seriously resulting in exacerbated symptoms, in some cases needing urgent care:

*“The last time I went for my cellulitis, I said it was blood circulation* < *..* > *she said, 'No, it's not. Just take some paracetamol.' I say it's painful, there's a lot of colour on my leg, the colour has changed, it is black. She said, 'No, it's fine, take paracetamol.' I take paracetamol, my leg keeps going black. It was spreading black, and then it was painful. One morning I went to the surgery* < *..* > *same doctor see me, she jumped from her chair, she said, 'What's happened to your leg?' I said, 'I showed you last time, you told me to take paracetamol* < *..* > *She phoned reception to send me to the general hospital. She couldn't believe how it had changed in a matter of two weeks.” (P22)*

Participants who experienced mistreated or undertreated multiple health concerns felt they were disbelieved and their experiences were discredited. Although some participants became proactive in pursuing necessary treatment and care, others were less assertive in articulating their dissatisfaction with the current care:

*“I get stuck with trying to explain health problems* < *..* > *I had pain in the chest, and I've gone to the doctors and been referred for a scan. It was more or less me insisting that I have a scan done* < *..* > *I felt like I'm asking for this referral where I don't really need it, and it's very reluctantly done. Anyway, I had this scan done* < *..* > *I was given a clean bill of health in November* < *..* > *Two months later in January* < *..* > *I have a heart attack. Now, it turns out I needed four stents put in.” (P5)*

In response, some participants taken up self-medication, others denied their health problems, a few felt defeated:

*“I ended up self-medicating* < *..* > *I started buying it from Turkey, and I started self-dosing, because I was that desperate, because my body was shutting down. I wouldn't leave the house for days. I could barely function* < *..* > *they [doctors] were very dismissive.” (P19)*

Participants reflections suggested that unless their physical or mental health issues turned into a *‘*crisis management’, support offered in a time of need was minimal and fragmented*.*

3. No consistent support for managing multiple LTCs

Another key concern expressed by many participants was around irregular and poorly coordinated access to support in primary and secondary care for dealing with MLTCs. Participants highlighted several responsible constraints namely, long waiting lists, delayed or absent follow up procedures and no continuity between departments. Naturally, this resulted in deteriorated symptoms causing long-term burden to participants and pressures on health systems:

*“There's a one/two-year waiting list to see a neurologist. You're lucky if you see a neurologist that actually knows of your condition* < *..* > *There's no continuity. There's no liaison. It's so depressing!” (P10)*

Following a prolonged delay, available support was still constrained as services had time restrictions, reduced face-to-face contact, ‘back and forth’ care between departments, incorrect referrals and lack of professional expertise in MLTCs. Having to continually navigate the challenges surrounding healthcare system was tiresome and added *“almost like another illness* < *..* > *trying to coordinate your own care when you can't do it because you have no true power in it.” (P2).* Participants felt let down*.* They believed that their efforts in trying to better themselves were not reciprocated and support-seeking was overall inconclusive:

*“You almost have to shout in order to get seen rather than someone actually going, 'Well, I can see what you're trying to do; let us help you.'* < *..* > *in regard to my diabetes, it was pretty much basically you need to go, lose weight, get your blood sugar levels down, which I've done, but off on my own back* < *..* > *I don't have any kind of support in that* < *..* > *you end up making our own decisions, whether they be right or wrong.” (P15)*

Participants recognised that healthcare professionals were under immense workload pressures and budget limitations; however, they were concerned that support has become reactive in solving short-term critical problems without adequate planning for supporting long-term chronicity of people with MLTCs like P + MH. Poorly coordinated access to health support was challenging and raised concerns about progressive health deterioration.

4. Medication worries and frustration with lack of alternatives

In addition to healthcare system related challenges, participants were worried about medication side effects linked to long term use and polypharmacy. Reflections showed that many participants were concerned about potential long-term damage to their bodies, such as for their bone density, and some also experienced serious impacts such as medication interactions leading to a mental health episode:

*“GPs have offered me antidepressants, which I've tried, and there are risks with epilepsy medication. I've tried them and I've had such severe side effects and I've felt so much worse and so unwell* < *..* > *I actually went to a doctor's appointment before COVID and did express that I was suicidal* < *..* > *and then nobody phoned me back. Nobody phoned to see if I was okay” (P2).*

Although for most, medication has enabled better coping with symptoms, particularly for those with chronic pain, solutions for managing medication-related worries were not adequately implemented. Participants wanted a change such as ‘come off’ the medication, reduce dosage or change to alternative medicine, however, this was unsuccessful. Some felt uninformed on appropriate treatment adherence. Others, however, wanted added alternatives such as non-clinical approaches like hydrotherapy, acupuncture and other options that can ease relying on analgesics:

*“I became quite desperate to find other ways to cope, other than painkillers* < *..* > *I take codeine regularly and I know that that could be addictive* < *..* > *I've also looked a lot into breathing techniques and that helps with the anxiety” (P13)*

Nonetheless, most of the alternative approaches had financial ties such as service costs, travel arrangements or other provisions that had exclusions on access, causing disappointment.

#### Theme Five. SPLW support pathway to address the needs of people with P + MH LTCs

This theme comprises two sub-themes linked to benefits and challenges in acquiring SPLW led support. When presented with a definition of SPLWs and explaining their scope of support, naturally, there was a mixture of responses; participants who knew and/or had previous experience with social prescribing, and those who have not heard of SPLWs, but shared reflections of how they envisioned this type of support could materialise to address the needs with P + MH LTCs.

1. Emerging challenges

This sub-theme is further categorised into three additional codes and is presented ahead of benefits to reflect the initial challenges such as those related to the awareness of available SPLW support and delivery related issues that delayed involvement and engagement with SPLW support.

- 1.1. Lack of awareness of Social Prescribing Link Workers: Who, Where, How?

When asked about pursuing support through SPLWs, a large portion of participants reported that they had no clear understanding around the presented concept, and have either never heard of SPLWs, heard it through a ‘word of mouth’ such as friends, seen an advert in a research study or seen a brief sign on “*a door with social prescriber written on it” (P15)*. As awareness played a key part, naturally, participants had minimal understanding on the extent of wide-ranging benefits, coordination of support or the process of referrals, including eligibility:

“*In terms of social prescribing, that's never been offered to me. I'm not sure if that would help* < *..* > *I don't know what kind of social prescribing things would be beneficial*.” (*P12).*

Those who were vaguely familiar with SPLW support, associated it with specific groups such older individuals, unemployed people and for targeting specific health concerns such as diabetes or weight loss. Participants were frustrated and therefore reiterated the importance of responsible parties ensuring better advertisement and communication for navigating SPLW support:

“people who might need it, or might benefit from it, need to be told it's there. <.. > otherwise, how do people know about it?” (P17)

However, when elaborated on the scope of SPLW role, participants were in agreement that this avenue of support could enable better management of MLTCs, could reduce isolation through participation in community initiatives such as befriending, and importantly could help participants feel equal and accepted rather than being seen as a patient.

- 1.2. Lack of appropriate training and skills

Another set of responses, particularly from participants who either received or attempted to seek SPLW related support, revealed gaps in Link Workers skillset. For example, in several cases participants left feeling underwhelmed with the extent of help that they were able to acquire from their SPLWs. After doing extensive research on finding out their representative SPLW, participants hoped for a personalised assessment of their needs and circumstances to help tailor avenues of support for tackling issues such as chronic pain, depression related struggles as well as sourcing support or consultation with completing applications such as ‘Personal Independence Payment’. Instead, participants were offered the option of being signposted to a GP and other general support agencies or not signposted at all under the assumption that participants were already proactive and explored their options. In turn, participants felt dismissed and believed that the offer of support was inadequate:

*“So I spoke to her [SPLW] about two weeks ago, and, again, shocking really. So she's lovely. Really nice lady, but ineffectual. 'Oh gosh.' She said, 'I don't really know how I'm going to help you. You seem to be doing…'Again, I get this all the time. You're already doing the things’* < *..* > *So I'm like, 'Look, okay, I've got a couple of things. Maybe you could look into it for me* < *..* > *It was like the whole role was reversed.* < *..* > *Oh my God, really? Is that really what's out there for people?” (P19)*

- 1.3. Lack of personalised and practical solutions

When discussing arrangements for applying SPLW referred support, participants shared a set of practical concerns that they felt needed to be addressed for this support pathway to make a positive impact. Namely these were:

- tailoring mode of delivery to accommodate participants’ symptoms exacerbations and the shifting motivation: *“You don't have the stimulus of lots of things going on around you, and that is quite tiring. So, actually going online is really useful for me* < *..* > *it could be hybrid so you could choose” (P1),*
- addressing financial implications and affordability of referred activities: *“You know I can't afford this £10 a go or whatever it is to go to” (P11),*
- improving timetabling of activities to accommodate individuals in full-time employment or with caring responsibilities: *“for example, the walking group from the GP, it tends to be during the day.* < *..* > *There isn't a six o'clock one, or an early morning one” (P15),*
- considering location and distance to activities: *“There are a lot of things to really consider, which has to do with the convenience and the transport for my location” (P21),*
- unifying resources for a consistent and accessible use: *“you need some sort of unified mechanism, even if it's done by an app* < *..* > *You go to one website, and they're going to tell you five things* < *..* > *it would be nice to think that there'd be a way by all of these things could be brought, essentially, under one umbrella” (P8).*

Another significant concern related to long-term support and how well SPLW support pathway was optimised to accommodate the chronicity of MLTCs. Participants reflected that most of the support they have received in the past was a one-off course or session that given short-term solutions without a plan for tackling exacerbations or access for continued support. Thus, arrangement of this nature was seen as necessary:

*“You could go years without it but somebody that you could pick up the phone and say, 'I'm not dealing well with this. Help.'* < *..* > *Maybe it's somebody like who could signpost you to services and could get you into services.* < *..* > *That is really empowering to have that, even if you never use it.” (P12)*

Additionally, others emphasised personal factors and called for SPLW led support to be tailored around age, gender and race to reflect specific interests, values and needs:

*“that form of trust would really come from your racial origin* < *..* > *if I should approach a diabetes nurse who is of my racial origin, he will be able to understand where my ethnicity and my background is coming from. Advise me properly on the food I should stop eating and how I should be eating* < *..* > *I really think those are some of the gaps that need to be more addressed.*” *(P21)*

Participants felt that referrals to group related activities with mixed socio-demographics was not compatible, and instead it was a barrier for opening up and being able to relate to others:

“For me, I enjoy an interaction where we are all more or less in the same—having quite similar demographics. I'm mainly talking of age group and also level in life and social status and all” (P20)

Tailoring support plan and arrangements to individual circumstances and priorities was essential for improving engagement with SPLW support and enabling person-level benefits.

2. Seeing the benefits

This sub-theme presents a set of grouped benefits of SPLW led support. As with earlier outlined sub-theme, this sub-theme holds responses from participants who engaged with SPLW support and those who envisioned how SPLW support could benefit them. Namely:

- arranging and/or delivering ‘soft’ preventative interventions and resources to address lifestyle, health and psycho-social related issues like loneliness, social isolation or motivation related struggles: *“I think social prescribing is an excellent idea.* < *..* > *They linked me in with the* < *..* > *RSPCA Centre. So, I used to go there and basically run the dogs.* < *..* > *So, that was a win win situation.” (P7*),
- facilitating group activities for purposes such as connecting like-minded individuals to build friendships, exchange ideas and learn about MLTCs: *“I think it [SPLWs] can really potentially create a real community feel around people* < *..* > *they just get a bit of interaction, some kindness, a friendly face and long-term relationships being built* < *..* > *encourage people to talk about having conditions” (P14),*
- helping patients to navigate health systems such as coordinating and linking up arrangements between different departments to manage diverse MLTCs: *“if there is a link person who can try and get the different professionalisms talking to each other. So in my case, trying to get neurology, rheumatology and mental health services so that there's a link person that can actually get communication between the three so that's there's coordinated support” (P2),*
- participants shared concerns that in-hospital support can feel intimidating and demanding, thus they believed SPLWs were placed in a strategically good position to offer help outside hospital environment that can empower patients and help them feel at ease: *“I think keeping it in the community is so important. It needs to be a bit detached from the hospital* < *..* > *you can speak completely openly* < *..* > *a lot of people with chronic conditions don't have a lot of trust in doctors and medical professionals in general, just because a lot of us have had so many bad experiences” (P13).*

The wide-ranging SPLW led benefits were positively appraised and were linked to improved health and wellbeing outcomes through social engagement in the community. However, some of these reflections were visionary rather than lived experience, thus participants’ expectations may not be aligned to implementable actions for SPLW role.

## Discussion

Findings from this qualitative study illuminate the lived experiences of adults with P + MH LTCs about their range of health and psychosocial needs, and outline how participants felt the SPLW role was equipped to support them. Findings indicate that the situation for individuals with P + MH LTCs encompasses serious challenges, and that the prospect of SPLW support pathways to mitigate at least some of those complexities may not be adequately realised in practice. Our study was able to identify three broad areas of concern related to accumulative and wide-ranging impact and unmet mental health needs; persistent unmet issues with access to care, delivery and coordination of health services; and poorly utilised efforts for social connectedness.

Firstly, our findings highlight that living with P + MH LTCs was a complex experience that centred around accumulative and multifaceted struggles and adversity spanning diverse contexts and settings. While similar observations have been noted in previous research with individuals with MLTCs who noted complex limitations of living with multimorbidity [12–14, 68], our study was able to take a closer look at issues such as those related to everyday functioning and mental health needs. Reflections suggest that P and MH LTCs shared uncertainty and instability in having to constantly adjust one’s life to the needs of LTCs. This loss of control over their health and wellbeing exacerbated symptoms of depression with other unhelpful consequences. It was however concerning that most participants had no clear trajectory for treating and supporting their mental health long-term, despite instances of mental health deterioration. Although, some participants have taken a proactive role in utilising diverse coping strategies, some of which were to support the needs of physical conditions, the impression is that mental health burden was downplayed and somewhat seen as a side effect to living with MLTCs. Our study observed that there is lack of adequate attention to the seriousness of people’s with MLTCs mental health as there was a tendency of ‘*Surviving but not Thriving’*, similarly observed in the Mental Health Foundation’s work [69], suggesting collective deterioration in nation’s mental health that has not improved. This observation is aligned to several recent reports that highlight a mental health crisis in the UK [70–72]. While there are emerging potential solutions such as low-intensity psychological interventions for treating depression and anxiety in people with LTCs [73, 74], evidence is limited and effectiveness is variable; pointing at the need for tailored interventions that would also consider wider socio-economic factors of those patients [75]. Thus, joined-up multi-disciplinary efforts with community assets such as SPLW led support may add this value for streamlining integrated support that is aligned to patients’ needs, circumstances and values. However, integrated partnership between SPLW with primary and secondary care requires further work and practical solutions. Particularly, as evidence on social prescribing programmes for mental health problems like depression and anxiety is conflicting, suggesting the need for comprehensive interventions underpinned by appropriate development processes, theory, co-design activities and effective evaluation processes [76].

Secondly, our findings resonate with previous work [9] that identified concerns about a lack of timely and coordinated access to care for older adults with MLTCs, questioning poor progress in applying a widely recognised recommendation of integrating health and social care through holistic, personalised and multidisciplinary models of care, and moving away from a single-disease solution. Findings in our study have confirmed similar concerns around isolated care and treatment of LTCs, and participants’ responses confirmed the need for a ‘whole-person’ approach. While this is not novel and has been recognised in the NHS Long Term Plan 2019 [44], it reiterates the scale of impact of this prolonged delay for integrated care that patients are facing daily. Our study has extended this finding and highlighted additional unproductive pattern of reactive treatment and care that prioritised short-term areas of concern over long-term proactive and preventative multiple illness planning. Findings demonstrated that reactive support has turned into a crisis management, that effectively dismissed participants’ expectations in addressing ‘root causes’ of P + MH LTCs and supporting planning for managing long-term chronicity. Our finding is aligned to a recently published NHS Confederation report [77] on ‘unlocking prevention in integrated care systems’ and shifting from treating ill-health to prevention.

Thirdly, our work demonstrated the importance of meaningful and compassionate social connectedness as an avenue of support for coping with wide-ranging physical and emotional needs of P + MH LTCs. The role of supportive social engagement has been widely evidenced, and it is a significant social determinant for improved wellbeing in people with MLTCs [78, 79], although our study identified diverse perceptions. For example, there was increasing interest and recognised value, but still limited uptake of community led social support groups due to hesitations around conceptual and practical set up and delivery of such groups. Furthermore, close social environments such as those related to family and friends provided stability and compassion, but simultaneously was a source of distress due to stigma, intolerance and lack of understanding around the impact for someone living with P + MH LTCs, but also due to prejudice related to participants’ sociodemographic factors. However, when inquired about social prescribing support through an SPLW, people identified value but there was a wide disengagement gap linked to non-intentional reasons. Given that this group of adults expressed an interest in community led activities that mirror social connectedness with peers, data in this study suggest that the capacity of SPLW support has not been applied accordingly for this group. There were missed opportunities for engagement and involvement due to mostly lack of awareness about the availability of this support model but also due to lack of personalised and holistic solutions to people’s with P + MH LTCs needs and circumstances. This observation may offer controversy given the widespread interest and uptake of SPLW led support. However, it highlights that there are ‘pockets’ of patient groups that have not used this avenue but could find it beneficial. This finding adds knowledge to a recent observation [37] that called for evidence clarification on groups that may be left out in social prescribing. Our data suggest the importance of broadening the community reach and engaging groups such as those from ethnic minorities, those facing socio-economic challenges and deprivation, men with experience of mental illness, younger adults and potentially other vulnerable sub-populations with LTCs who are less informed or less likely to engage with support avenues such as those not linked to mainstream health systems.

### Implications for research and practice

Our study findings offer several implications that can inform further research, and several areas of practice. For example, this group reported experiences of intolerance and stigma from diverse social environments on sensitive issues of living with P + MH LTCs and unmet mental health needs. It highlights the need for acceleration in public awareness to counter stigma on mental health and chronic conditions, as aligned to recent reports [72, 80]. In particular, large portion of participants in this study were employed and had experiences of unfair pressures in navigating demands between their chronic illnesses and work commitments, without considerate support. Similar to our observations, Sand [68] found that people with multimorbidity feel strongly affiliated to their social identities like ‘being a worker’ and can experience distress when the valuable identity is compromised. We suggest that interventions like SPLW support may be strategically placed to pursue active advocacy role in employment tailored social prescribing programmes and/or public health campaigns to engage diverse stakeholders on sensitive issues of living with P + MH LTCs to support affected individuals in addressing their health and social needs while in employment. However, to date evidence on social outcomes like employment is limited [39]; and this may have been out of scope and, due to capacity and expertise inconsistencies, difficult for SPLWs to implement, particularly as SPLW support pathway is diverse and may not be equally resourced to accommodate this issue.

In addition to broader themes, we shed light on other less discussed, but complex topics that may require closer unpicking to understand the potential relevance of SPLW support. For instance, there were reports on a) increased alcohol use in adults with P + MH LTCs, b) lack of awareness and support on issues related to men and women reproductive health, such as male erectile dysfunction or gynaecological conditions, in the context of living with MLTCs, and c) references to suicidal thoughts as a result of the burden from P + MH LTCs. Our observations mirror the wider literature on the need for targeted mental health training for SPLWs to be able to respond to traumatic experiences and other mental health concerns [26, 32].

Furthermore, findings in this study demonstrated that there is a wide unawareness and uncertainty about social prescribing support in the community that may have contributed to this group’s low levels of engagement. It highlights that there is a need for better promotion and navigation of available SPLW services particularly, to improve coordinated access to community led social support groups. This study population has recognised the value in community assets for their health and wellbeing, particularly those that mirror social connectedness with peers, however, due to lack of advertisement and unclear utilisation of SPLW led support in the community, opportunities for targeted support services and activities have mostly been missed. It is, therefore, important that efforts are scaled up to actively involve and engage communities in knowledge sharing, intervention development and production activities and participation in creative community initiatives that can increase conceptual and practical awareness of social prescribing, but also achieve a greater community engagement and collaboration on issues that affect the target group [26, 27, 37].

Altogether, we found potential obstacles in SPLW role for supporting adults with needs in P + MH LTCs and identified areas where SPLW involvement could add valuable layer of support for this group. These findings have set the basis for the next stage in our research that will extend this line of inquiry and will examine experiences and perspectives of SPLWs through focus groups to strengthen current knowledge on SPLW support as an avenue for managing issues around P + MH LTCs, and to enable guidelines recommendations for improvement.

### Strengths and limitations

Our study utilised a rigorous and inclusive recruitment strategy focused on addressing gaps in evidence related to a previous lack of clarity and diversity in samples with MLTCs. Given that much of the understanding around SPLW led support centres around conditions like diabetes, knowledge translation to other LTCs is limited and may not represent the multi-layered needs from groups that are less involved in research. While our findings are not necessarily generalisable beyond included LTCs in the study, they offer insight into the needs of adults with particularly diverse MLTCs. The cohort in this study had experience with P + MH LTCs spanning conditions related to bowel, chronic pain, gynaecological, cardiovascular, metabolic, respiratory, neurological, rheumatoid, sensory, thyroid and other physical LTCs together with mood disorders. This broad inclusion of diverse LTCs extends knowledge beyond common clusters of conditions and highlight shared commonalities that can better inform stakeholders in tailoring SPLW support for patients with P + MH LTCs. We have also extended our efforts in recruiting participants from communities that are less involved in research and may not have their experiences voiced, such as certain ethnic minorities, as well as groups from socio-economically challenged backgrounds (e.g., people accessing food banks). It is with caution that we make these observations as the socioeconomic status of participants cannot be fully determined since income was not assessed, thus, it may not be capturing a range of socioeconomic status.

A recently co-produced NHS practice guide for engaging underrepresented groups in research reported the link between health disparities and seldom heard groups, meaning that those with the highest burden of illness have lowest participation and engagement in research [59]. This suggests urgency to tailor recruitment strategies that would support mindful and culturally sensitive involvement and engagement of diverse communities in research. We observed that although more targeted outreach engagement is necessary to involve other less represented communities such as South Asians, our recruitment activities through VCSE and community assets have benefited several areas such as: a) helped to raise awareness of social prescribing in the community; b) offered a platform for participants with diverse MLTCs to voice their needs; and c) added more detailed view on sociodemographic information of individuals with P + MH LTCs. We have treated any data-sociodemographic relationship with care to ensure that participants’ identities are not identifiable or are linked to specific individuals. This in turn may limit the opportunity for teasing out specific relational traits but instead it offers wider observations on categories like age and gender groups, employment, education and living arrangements together with a numerical representation of conditions per sample. We recognise that future research should also collect information about participants’ income as essential indicator of socioeconomic status.

Lastly, the geographical reach in this study spanned the Wessex region, which may constrain the generalisability to a wider audience. It brought attention to communities affected by P + MH LTCs who shared commonalities in experience not exclusive to the region, and it also highlighted complexities around the SPLW utilisation that may be applicable nationally and further afield. Identified knowledge is also important for informing decision making of local commissioners and primary care networks, but also for VCSE that facilitate some of the social prescribing programmes on areas that need improvement.

## Conclusions

This study has shed light on important community issues and lays out an important trajectory for future work. Our study findings demonstrate that living with multiple conditions like P + MH LTCs can be a constantly shifting experience, with competing multi-layered needs and demands; and that there is a clear priority for adequately integrated health and care systems with support avenues like SPLW to ease growing health and psychosocial pressures. More targeted interventional work is necessary to widen the reach and potential relevance of SPLW services, particularly amongst sub-populations in-need with experience of P + MH LTCs.

## Supplementary Information

Supplementary Material 1.

## Data Availability

The dataset generated and analysed during the current study are not publicly available due to the confidential nature of transcript data but are available from the corresponding author upon reasonable request.

## Abbreviations

P + MH LTCs: Physical and Mental Long-Term Conditions
MLTCs: Multiple Long-Term Conditions
SPLW: Social Prescribing Link Worker

## Footnotes

1 ’Sectioned’ connotes detention under the provisions of the Mental Health Act (involuntary admission)’.
