# Supplementary material for: Understanding the potential role of Social Prescribing Link Workers in supporting identified needs of people with physical and mental long-term conditions: a qualitative study
Source: BMC Prim Care. 2025 Nov 19;26:372. doi: 10.1186/s12875-025-02990-z (PMC12628938; doi:10.1186/s12875-025-02990-z)
Supplement: Supplementary file 1 — Supplementary Material 1. [file 12875_2025_2990_MOESM1_ESM.docx]

Appendix 1 Interview schedule

**Semi-structured interview schedule**

I appreciate that you have accepted our appointment today. Thank you very much.

My name is Skaiste Linceviciute, I am a Research Fellow at the University of Southampton.

As you could read in the information sheet, in this research, we are interested in understanding your health and non-health related needs and experiences when living with physical and mental health long-term conditions (LTCs). Also, we would like to hear your perceptions about social prescribing link workers for supporting people with physical and mental LTCs, and to better understand your views and experiences with this (if any).

Please know that there are no right or wrong answers. We are keen to hear your views and experiences in your own words. You are free to discuss your perspectives openly during the interview. From time to time, I might ask you for further clarification to help me to understand your responses.

I would like to reassure you that this interview is confidential. We will pseudonymise personal identifying information that you provide during the interview, including your own name, so you will not be identifiable. The answers that you provide will only be shared with the research team and the transcription service approved by the university.

I would like to request your permission to audio-record this interview to help facilitate the transcription and analysis of the data during the course of this project.

Before we begin, I would like to ask you to reconfirm your consent to taking part in this study. Do you consent to taking part in this study?

Do you have any questions before we begin?

**INTRODUCTION POINTS**

- Introduce yourself - the interviewer
- Remind participants about the aim of this research study and thank them for agreeing to take part in the interview
- Talk about the purpose of the interview and how the information will be used
- Remind participants about the opportunity to pause, skip questions, ask the interviewer to clarify any concerns as well as opportunity to withdraw
- Introduce discussion topics

**Any questions?**

Participants will be asked to introduce themselves for the purpose of the recording. The interviewer will also gather sociodemographic information such as age, gender, marital status, ethnicity group, education level, employment status, area postcode, living alone or with somebody else.

***Understanding the needs and experiences of living with physical and mental health long term conditions***

1. Please tell me about yourself: How would you describe your health in your everyday life?
2. Tell me about your conditions: What it is like living with physical and mental health long term conditions? How do you manage these?
3. What role does/ do your long term condition(s) play in your life? If it/ they affect(s) your life, please tell me how it is affected. Please consider sharing examples to illustrate your experiences.
4. Thinking about health and non-health related needs of living with physical and mental health long term conditions, what are your ongoing needs?

*Prompts*: **health** (e.g., improve fitness, help to manage pain, help to manage tiredness, reduce isolation, reduce anxiety and improve coping strategies, improve motivation) and **non-health** (e.g., improve skills in using online materials/ internet, improve financial circumstances, receive housing advice, have peer support and build friendships, address transport issues, try new hobby)

1. Tell me about the possible barriers and enablers that play a role in the management of your physical and mental health long term conditions? If possible, please share examples to illustrate your experiences.

*Prompts*: **barriers** (e.g., lack of motivation/ willpower, fatigue that interferes with everyday activities, financial constraints, inadequate communication and support from healthcare providers, medication side effects) and **enablers** (e.g., support groups, WhatsApp groups with other sufferers for support and advice, clear illness management plan, community support, charities).

***Types of support for better living with physical and mental health long term conditions***

1. When thinking about support in your daily life with your long term conditions, what are the types of support that you might or do have?

*Prompts:*

- 1. If you receive support, please share examples and experiences of the support that you are receiving (e.g., part of a support group or a management programme, online courses, receive support from family/ friends, voluntary organisations, healthcare professionals/ NHS).

a 1. Please consider commenting if the types of support that you receive are useful and are there any gaps that could be improved?

- 1. If you do not receive any support, please consider what support would you like to have?

b 1. If you have tried to access support in the past and/ or struggled with it (e.g., not knowing where to begin searching for information, fear of prejudice), what do you think could be done differently about accessing support for people living with physical and mental health long term conditions?

1. When thinking about support from various service providers to help people with physical and mental health long term conditions, please tell me if you have you ever heard about ‘social prescribing link workers’?

*Prompts*:

Social Prescribing Link Workers are also known as community connectors, who are facilitators that connect individuals to sources of support within community to help address diverse needs of people living with physical and mental health long term conditions)?

- 1. If yes, please tell me about your experience with this type of support.

a 1. What role does the Social Prescribing Link Worker play in supporting your needs of living with physical and mental health long term conditions?

- 1. If not, please tell me if you would consider this type of support beneficial to you and your needs.

b 1. Please consider what type of support you would like to have to address your needs

1. Tell me your views about how support for people living with physical and mental health long term conditions could be improved.
2. Please tell me if you have any suggestions that you think are important when seeking support for people with physical and mental health long term conditions.
   1. Do you have any other comments or suggestions?

Appendix 2 Participants’ description

| Participant Number | Description |
| --- | --- |
| P1 | Female, 40-49,  LTCs: Anxiety, Depression, Metabolic, Neurological. |
| P2 | F, 50-59,  LTCs: Anxiety, Depression, Chronic Pain, Neurological. |
| P3 | F, 20-29,  LTCs: Depression, Respiratory. |
| P4 | Male, 50-59,  LTCs: Depression, Respiratory. |
| P5 | M, 50-59,  LTCs: Anxiety, Depression, Bowel, Chronic pain, Rheumatoid, Metabolic, Heart. |
| P6 | F, 50-59,  LTCs: Anxiety, Heart. |
| P7 | F, 50-59,  LTCs: Depression, Chronic Pain. |
| P8 | M, 40-49,  LTCs: Anxiety, Depression, Chronic Pain, Neurological, Rheumatoid. |
| P9 | M, 40-49,  LTCs: Depression, Chronic Pain, Neurological, Heart. |
| P10 | F, 70-79,  LTCs: Anxiety, Depression, Chronic Pain, Neurological. |
| P11 | M, 50-59,  LTCs: Depression, Chronic pain, Other (Long-Covid). |
| P12 | F, 50-59,  LTCs: Anxiety, Neurological. |
| P13 | F, 20-29,  LTCs: Anxiety, Gynaecological, Chronic Pain. |
| P14 | F, 30-39,  LTCs: Anxiety, Depression, Rheumatoid. |
| P15 | M, 50-59,  LTCs: Anxiety, Metabolic, Neurological. |
| P16 | M, 60-69,  LTCs: Depression, Metabolic, Sensory, Neurological. |
| P17 | F, 70-79,  LTCs: Anxiety, Depression, Neurological. |
| P18 | F, 40-49,  LTCs: Depression, Sensory. |
| P19 | F, 50-59,  LTCs: Depression, Thyroid, Chronic Pain, Neurological, Gynaecological. |
| P20 | M, 30-39,  LTCs: Depression, Metabolic. |
| P21 | M, 20-29,  LTCs: Depression, Metabolic. |
| P22 | F, 40-49,  LTCs: Depression, Heart, Neurological, Chronic Pain. |
| P23 | F, 30-39,  LTCs: Depression, Rheumatoid. |
